# Transcription enhancers as major determinants of SV40 polyomavirus growth efficiency and host cell tropism

**DOI:** 10.1099/jgv.0.000487

**Published:** 2016-07-01

**Authors:** Katharina Schmidt, Simon Keiser, Viola Günther, Oleg Georgiev, Hans H. Hirsch, Walter Schaffner, Tobias Bethge

**Affiliations:** ^1^​ Institute of Molecular Life Sciences, University of Zurich, CH-8057 Zurich, Switzerland; ^2^​ Transplantation & Clinical Virology, Department of Biomedicine, Petersplatz 10, University of Basel, CH-4009 Basel, Switzerland; ^3^​ Infectious Diseases & Hospital Epidemiology, University Hospital Basel, Petersgraben 4, CH-4031 Basel, Switzerland

**Keywords:** polyomavirus NCCR rearrangements/polyomavirus pathology/viral cell tropism/viral host cell preference/recombinant virus/organ specific infection

## Abstract

The non-coding control region (NCCR) of polyomaviruses includes the promoters for early and late genes, a transcription enhancer and the origin of DNA replication. Particularly virulent variants of the human pathogens BKPyV and JCPyV, as well as of simian virus 40 (SV40), occur *in vitro* and *in vivo*. These strains often harbour rearrangements in their NCCR, typically deletions of some DNA segment(s) and/or duplications of others. Using an SV40-based model system we provide evidence that duplications of enhancer elements, whether from SV40 itself or from the related BKPyV and JCPyV, increase early gene transcription and replicative capacity. SV40 harbouring subsegments of the strong cytomegalovirus (HCMV) enhancer replicated better than the common ‘wild-type’ SV40 in the human cell lines HEK293 and U2OS. In conclusion, replacing the SV40 enhancer with heterologous enhancers can profoundly influence SV40’s infective capacity, underscoring the potential of small DNA viruses to overcome cell type and species barriers.

The ever-growing family of polyomaviruses includes more than a dozen distinct members detected in humans (Lim *et al.*, 2013; Yu *et al.*, 2012). Of these, BKPyV and JCPyV, together with the carcinogenic Merkel cell polyomavirus (MCPyV), are the best-characterized ones. BKPyV and JCPyV are known to infect a large part of the human population worldwide (Egli *et al.*, 2009). They usually remain symptomless, but both of them can cause severe diseases in immunocompromised individuals including organ transplant recipients. BKPyV causes BKPyV-associated nephropathy and haemorrhagic cystitis, while JCPyV causes progressive multifocal leukoencephalopathy. The simian virus 40 (SV40) is a close relative of BKPyV and JCPyV and replicates particularly well in kidney cells of Old World monkeys, including the rhesus monkey and African green monkey (Butel & Lednicky, 1999). The genome of all polyomaviruses is organized as a circular double-stranded DNA of ~5 kb with a non-coding control region (NCCR) located between the divergently transcribed units of early and late genes. The NCCR includes the early and late promoters, the transcription enhancer and the origin of DNA replication. Incidentally, the SV40 enhancer was the first enhancer to be discovered and was, thus, the first example of this essential class of eukaryotic regulatory elements (Banerji *et al.*, 1981; Moreau *et al.*, 1981; Schaffner, 2015). Despite its conserved function, the NCCR/enhancer region is the most variable segment among polyomavirus genomes and can evolve quickly. Over time, evidence has accumulated that this variability can affect host cell preference (Couture & Lehman, 1993; Katinka *et al.*, 1980; Ondek *et al.*, 1987; Rochford *et al.*, 1987; Schirm *et al.*, 1987; de Villiers *et al.*, 1982; White *et al.*, 2009). The archetypal, commensal forms of BKPyV and JCPyV do not contain repeats in their NCCRs, but duplication and/or deletion of sequences turned out to be a hallmark of particularly virulent patient isolates and of laboratory strains of BKPyV (Bethge *et al.*, 2015; Gardner *et al.*, 1971; Gosert *et al.*, 2008; Seif *et al.*, 1979; Sundsfjord *et al.*, 1990; Watanabe *et al.*, 1984) and JCPyV (Gosert *et al.*, 2010; Padgett *et al.*, 1971). Importantly, it has also been suggested that NCCR rearrangements in JCPyV and BKPyV help the virus to overcome restrictions of cell type specificity and, thereby, contribute to the spread of pathology. Similarly, the archetypal SV40 from monkey isolates does not contain direct repeats but the laboratory strain commonly referred to as ‘wild-type’ has two tandem copies of a 72 bp enhancer segment (Ilyinskii *et al.*, 1992; Lednicky & Butel, 1997; Lednicky *et al.*, 1998; Newman *et al.*, 1998). Such NCCR rearrangements were suspected to duplicate activating sequences and remove inhibitory ones. Interestingly, the genuine SV40 enhancer can be substituted with enhancers from unrelated viruses or from cellular genes, as was shown by the so-called ‘enhancer trap’, a selection system that utilizes an SV40 genome lacking the 72 bp repeats and adjacent enhancer sequences to regain infectivity by incorporating heterologous enhancer-active DNA segments (Günther *et al.*, 2012; Weber *et al.*, 1984). In the current work we have focused on the enhancer segments of SV40, BKPyV, JCPyV and of human cytomegalovirus (HCMV) for their ability to alter the host range of SV40, thus expanding on preliminary findings that a synthetic enhancer assembled from transcription factor binding sites can facilitate SV40 early gene expression and DNA replication in human embryonic kidney-derived cells (Günther *et al.*, 2012). These studies are important since dual infections involving BKPyV, HCMV and SV40 have been reported in immunosuppressed transplant patients (Li *et al.*, 2002; Nada *et al.*, 2005). Moreover, co-infections and subsequent rearrangements can contribute to a broader cell and host tropism, at least *in vitro* (Henriksen *et al.*, 2014; Kristoffersen *et al.*, 1997; Myhre *et al.*, 2010), and possibly support interaction, adaptation and pathology of polyomaviruses in new species including humans (Rinaldo & Hirsch, 2013).

First we tested two major forms of SV40: the archetypal one with only one 72 bp enhancer segment and the laboratory 776 strain with two copies. Since the SV40 archetype grew almost as fast as the wild-type in monkey kidney CV-1 cells (not shown), both viral forms were tested by competition in a co-transfection experiment. Even if the archetype was initially present in fourfold excess, it was swiftly overtaken by the laboratory strain such that in a second round of mixed infection, seeded with an aliquot of culture supernatant from the first round, only the laboratory strain with two 72 bp repeats was detectable (Fig. 1a). This result indicates that duplication of an active subsegment can confer a robust competitive advantage over the shorter form, despite the fact that there is, in principle, redundancy in the information content of enhancers (Schaffner *et al.*, 1988).

**Fig. 1.**
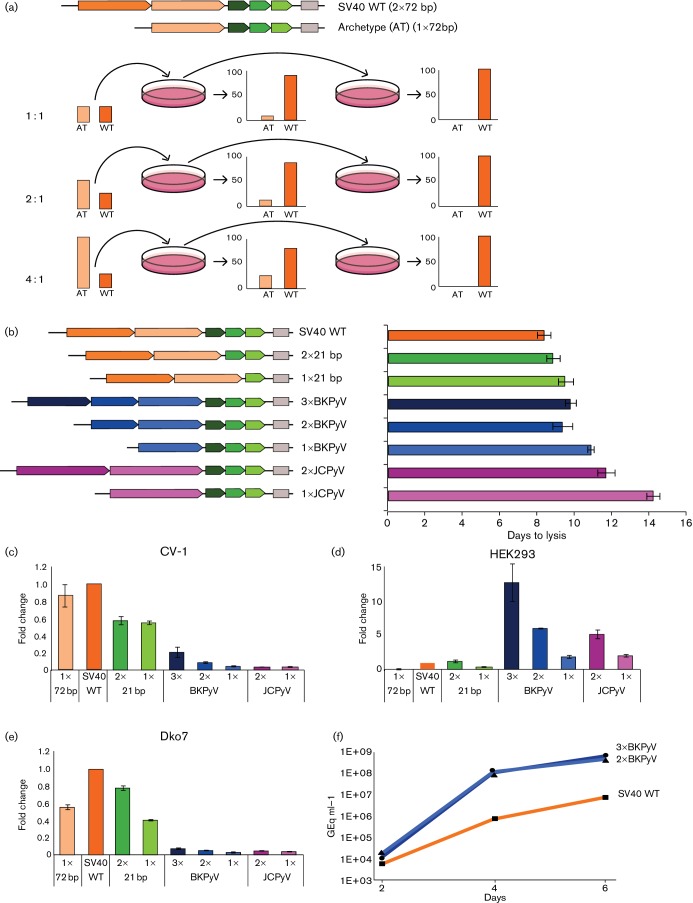
Effect of duplication of enhancer segments on viral growth. (a) Growth competition between SV40 harbouring one 72 bp enhancer segment (archetype) vs two 72 bp segments (‘wild-type’ lab strain). Top, schematic of the control region for early transcription in the two strains tested. The cloned SV40 genomes containing 1×72 bp (archetype; light orange bar) or 2×72 bp (wild-type; deep orange) were liberated by *Bam*HI cleavage from the vector plasmid, mixed in ratios of 1 : 1, 2 : 1 and 4 : 1 (100+100 µl, 200+100 µl, 400+100 µl) and transfected into CV-1 monkey kidney cells by the calcium phosphate method. After the spread of infection, viral DNA was extracted from the cells by the alkaline precipitation–neutralization method, digested to distinguish the fragents containing 1×72 bp vs 2×72 bp, run on an agarose gel and quantified. A 300 µl portion of centrifuged medium supernatant was used to infect a new dish of CV-1 cells and the procedure repeated. Upon re-infection, cell lysis and analysis, only bands with the double 72 bp repeat were visible. (b) Effect of single and multiple repeat elements on SV40 growth. The three 21 bp repeats [green; SV40 genome position (pos.) 41–103] with binding sites for Sp1 transcription factor are considered part of the early promoter, rather than the enhancer. Diminished copy numbers of this segment (2×, 1×) also affect virus growth. The SV40 72 bp repeats (orange; pos. 107–250) were replaced by three, two, or one repeat derived from the BKPyV ‘Dunlop’ lab strain (blue; pos. 149–333); SV40 recombinants with one or two 98 bp segment from JCPyV were also generated (purple; pos. 12–206). For each construct, three independent transfection mixes were prepared to transfect in parallel three dishes of CV-1 cells. Dishes were regularly inspected for the progress of the infection; the day when half of the cells were dead, with a shrunken nucleus and ready to detach from the dish, was taken as the endpoint (shown on the righthand side). The enhancer segments correspond to those of virus strains 776 (SV40) (Fiers *et al.*, 1978; Reddy *et al.*, 1978), Dunlop (BKPyV) (Seif *et al.*, 1979) and Mad-1 (JCPyV) (Frisque *et al.*, 1984). (c–e) Transcript levels induced by different copy numbers of enhancer repeats. The enhancer region of each construct shown above was subcloned into the OVEC reporter gene (Westin *et al.*, 1987), and transfected together with a reference gene (OVEC-REF) into the three indicated cell lines (monkey kidney CV-1, human embryonic kidney-derived HEK293, and mouse fibroblast-type Dko7). Two days later, RNA was extracted and quantified by S1 nuclease mapping. Transcript levels with SV40 wild-type repeats were set to 1. The colour code of the bars is as in (b). The error bars, showing sem, are derived from two independent transfection experiments. (f) Recombinants with two or three enhancer repeats from BKPyV outperform SV40 if tested for virus production in HEK293 cells. Cells were transfected with virus genomes liberated by *Kpn*I digestion from the vector plasmid. Two, four and six days after transfection, supernatant samples were collected, and cell debris was removed by centrifugation. Quantification of DNaseI-protected SV40 genomes was carried out by quantitative PCR (TaqMan) on an ABI 7500 (Thermofisher) as described (McNees *et al.*, 2005). Three parallel quantifications of each sample yielded essentially identical results; thus error bars are not visible. Virion production was verified by infection of CV-1 cells (not shown). Virus growth is in agreement with the poor activity of the SV40 enhancer in these human cells (Günther *et al.*, 2012) and the high activity of the BKPyV segments (d).

Besides the 72 bp segment, which occurs in one or two copies, the SV40 promoter contains three imperfect repeats of 21 bp, each harbouring two binding sites for the transcription factor Sp1. Viral growth efficiency in CV-1 cells was reduced when only one or two copies of this 21 bp segment were present [Fig. 1b; see also Barrera-Saldana *et al.* (1985)]. In the same setting we also tested NCCR enhancer segments that had become repeated in strains of BKPyV and JCPyV upon their adaptation to growth in cell culture. SV40 lacking its own 72 bp repeats was reconstituted with one or more of these segments, transfected into CV-1 cells, and the efficiency of virus propagation was monitored. As is evident in Fig. 1b, the time to lysis was less for viruses containing repeated enhancer segments, in support of the model that this usually results in faster virus growth. The relatively weak activity of archetypal, non-repeated enhancers in SV40, BKPyV and JCPyV must nevertheless be of biological relevance - it probably helps these viruses to remain under the radar in immunocompetent hosts.

We also tested the different repeat numbers for their transcriptional efficiency. For this we used the versatile globin gene-based reporter system (Westin *et al.*, 1987). Enhancer segments were inserted upstream of the reporter’s TATA box and transcript levels determined by the S1 nuclease assay. Note that reporter and reference genes do not replicate in transfected cells, which precludes possible confounding effects due to template copy number variation. The assay also indicates the location of the transcription start from the genuine reporter cap site and thus would reveal an altered transcription initiation, for example from within the enhancer. Three cell lines were tested: monkey CV-1, human embryonic kidney-derived HEK293, and mouse fibroblast-type Dko7 cells (Fig. 1c–e). In CV-1 cells, the number of 21 bp promoter segments and 72 bp enhancer SV40 repeats correlated with transcriptional activity; the BKPyV and especially the JCPyV repeats were weakly active (Fig. 1c). Of note, while transcriptional activity is clearly correlated with viral growth, the relationship did not appear to be linear; SV40 with BKPyV repeats grew better in CV-1 cells than would be expected from the transcript quantification (Fig. 1b vs c). This might indicate a contribution of SV40 sequences in the viral growth assay (see also below). In HEK293 cells, the BKPyV enhancer repeats were highly active and repeat numbers correlated well with transcriptional activity; even a single copy outperformed the SV40 enhancer, which was only poorly active in these cells (Fig. 1d). Given the strong activity of BKPyV enhancer repeats in HEK293 cells, we wondered whether SV40 with two or three BKPyV repeats would be able to productively multiply in these cells. To this end, cloned viral genomes were liberated by restriction digestion and transfected. After two, four and six days, the supernatant medium was analysed for viral load. As shown in Fig. 1f, in human cells the SV40-BKPyV recombinants yielded almost two orders of magnitude more virus than the wild-type SV40 (2×72 bp), again indicating that the repeated enhancer segments of the (rearranged) BKPyV Dunlop strain work well in HEK293 cells. Our findings also suggest that the BKPyV enhancer cooperates well with the SV40 early promoter. In this context we note that archetypal BKPyV neither produces T antigen nor replicates in HEK293 cells unless large T antigen is provided *in trans* in modified, so-called HEK293TT cells (Broekema & Imperiale, 2012). Furthermore, the archetypal BKPyV early promoter is weak even in natural host cells like human kidney RPTECs – most likely because it contains only a single Sp1 site compared to six in the SV40 promoter (Bethge *et al.*, 2015).

In the next set of experiments, we tested the ability of the enhancer of the immediate early-1 gene of HCMV for its ability to promote SV40 growth, relative to the performance of the genuine SV40 enhancer. The HCMV enhancer, unlike the one of SV40, is strongly active in a great variety of cells and thus widely used in biotechnology for protein production in mammalian cells. A genomic HCMV segment harbouring the enhancer was fragmented by sonication, mixed with an enhancerless linear SV40 genome and transfected into CV-1 cells. Using the ‘enhancer trap’ selection system (Fig. 2b) (Boshart *et al.*, 1985; Günther *et al.*, 2012; Weber *et al.*, 1984), we obtained ten chimeric SV40-HCMV viruses, containing independent, but overlapping, enhancer inserts of various lengths and orientations relative to early transcription (Fig. 2c). In separate infections of CV-1 cells, most of the clones grew well but the one with the shortest HCMV insert (no. 10) performed poorly in this and other experiments. In the monkey cells, however, SV40 multiplied faster than the most efficient SV40-HCMV recombinant clone 7 (Fig. 2d). To determine whether any of the SV40-HCMV clones could propagate in human cells, a serial competition experiment (similar to the one in Fig. 1a) was done with the following four human cell lines: HEK293 (embryonic kidney-derived), U2OS (osteosarcoma), HepG2 (hepatoma) and Hela (cervix carcinoma); monkey CV-1 cells were used as a control. We used HEK293, rather than the derived, widely used HEK293 T cells because the latter constitutively express SV40 T antigen, which would have confounded the results. An equimolar mix of recombinants was ensured by quantifying viral genomes from lysate supernatants. From each cell type, two dishes were infected: one received only the ten SV40-HCMV recombinants; in the other one SV40 was also included. After seven days, cells and supernatants were harvested and processed as indicated in Fig. 2e. DNA bands from the fourth round of selection were cloned and individual colonies were sequenced. Selection in HepG2 and HeLa cells was discontinued after the first round because no viral DNA was detectable. Interestingly, even though all of the recombinants harboured overlapping segments of the HCMV enhancer, the competition indicated some cell type preferences (Fig. 2f): in HEK293 cells, clone 6 emerged as the predominant virus; clone 1 was strongly represented in U2OS cells but played at the most a minor role in HEK293 cells. Unlike the situation in monkey CV-1 cells, SV40 was not able to compete in these human cells with the more efficient chimeric SV40-HCMV clones. Also in another experiment with human embryonic retinoblast-derived 911 cells, clones 5, 6, 7 and 9 replicated faster than SV40 (not shown). Thus, with the heterologous HCMV enhancer, SV40 readily multiplied in three of the five tested human cells (HEK293, U2OS and 911), in line with the concept that the transcription enhancer is a major determinant of SV40’s cell type and species specificity.

**Fig. 2.**
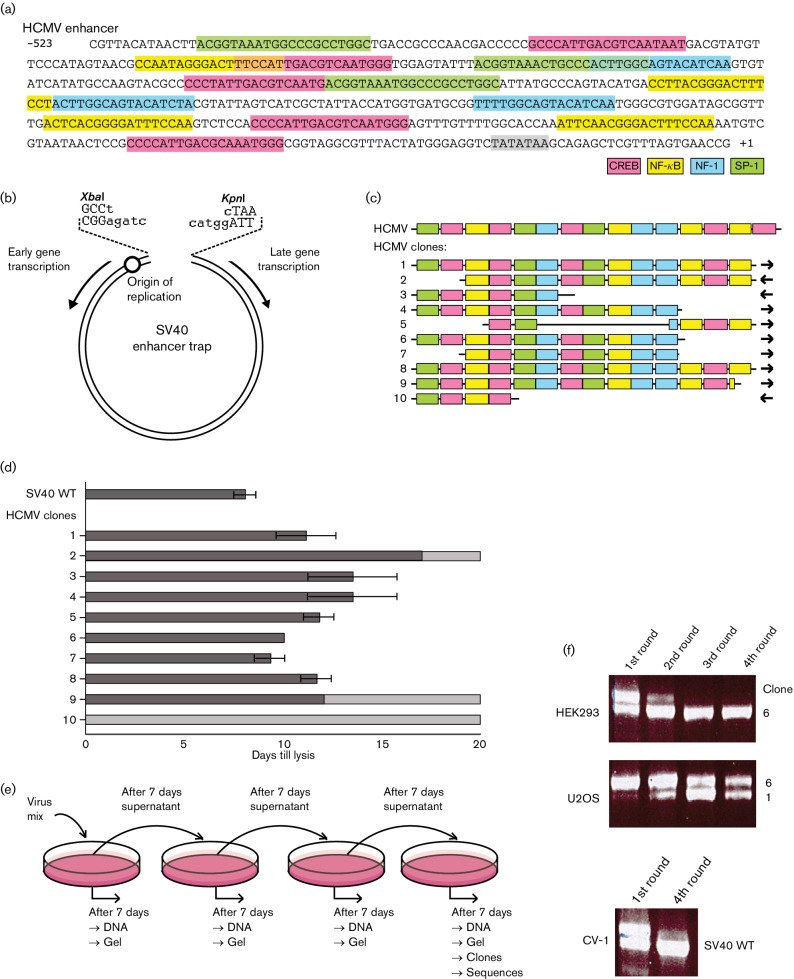
Overview of the SV40 enhancer trap and SV40-HCMV recombinants. (a) The enhancer of human cytomegalovirus (HCMV) (21). A conspicuous feature of this enhancer are multiple copies of binding sites for each of the transcription factors CREB (pink), NF-κB (yellow), NF-1 (blue) and Sp-1 (green). The TATA box of the HCMV immediate early-1 promoter is shaded in grey. (b) The SV40 enhancer trap. A defective SV40 genome lacking the enhancer (deletion between positions 99 and 294) can incorporate enhancer-active DNA segments inside the transfected host cell, which restores virus growth (Weber *et al.*, 1984). (c) Schematic view of the ten different HCMV inserts in SV40-HCMV recombinant clones. Note that in clone 5, a large internal region is deleted; clones 1 and 8 are slightly shorter than the full-length enhancer segment and differ from each other at their junctions with SV40 DNA. For simplification, spacers between the binding sites for the above-mentioned transcription factors (in a) are omitted. Arrows indicate the orientation of the inserts within the SV40-HCMV recombinants. (d) Growth of SV40-HCMV recombinants in monkey cells. The ten recombinant viruses were grown individually on CV-1 cells, and the day when half of the cells were dead was taken as the endpoint. Dark grey: time to 50 % lysis of cells. Light grey up to 20 days: no clear cytopathic effect in at least one dish (clone 10 nevertheless produced enough virus for the competition experiment shown in e and f). The error bars, indicating sem, are derived from infections done in duplicate dishes. (e) Growth competition of SV40-HCMV recombinant clones. An equimolar mix of SV40-HCMV recombinant viruses from infected cell supernatants was used as starting material to co-infect human HEK293 and U2OS cells, as well as monkey CV-1 cells. After four rounds of serial infection whereby 100 µl aliquots of supernatant were transferred to a new dish, viral DNAs were isolated by the alkaline precipitation/neutralization method and analysed further. (f) Agarose gel electrophoresis of viral DNAs. After each round of competition selection, viral DNAs were recovered for fractionation by agarose gel electrophoresis. After the fourth round, DNA from the gel bands was isolated, cloned and sequenced. In HEK293 cells clone 6 became dominant at the expense of the others, whereas in U2OS cells clones 1 and 6 were co-dominant. In CV-1 cells, where DNA was only analysed after the first and the fourth round, SV40 wild-type had outcompeted the SV40-HCMV recombinants by the end of the selection procedure.

Taken together, these results underscore the functional plasticity of the enhancer elements in polyomavirus NCCRs, which can subvert the replicative restriction occurring in host cell types not primarily infected (secondary host cell tropism). The *in vitro* experiments here suggest that in the absence of a functional immune control, rearrangements including enhancer recombinations in the case of co-infections might not only contribute to organ pathology, but enhance cross-species transmission. Here, transplant patients might represent a critical, underestimated mixing vessel.
